# Effects of exercise on balance in people with Parkinson’s disease: an umbrella review of systematic reviews and meta-analyses of randomized controlled trials

**DOI:** 10.3389/fpsyg.2026.1939213

**Published:** 2026-10-13

**Authors:** Yuning Yuan, Hui Ran, Youyou Song, Shiyan Xu, Kangcheng Chen

**Affiliations:** 1 College of Physical Education, Chuzhou University, Chuzhou, China; 2 Department of Endocrinology, Xinhua Hospital Affiliated to Shanghai Jiao Tong University School of Medicine, Shanghai, China

**Keywords:** AMSTAR-2, balance, exercise, GRADE, meta-analysis, Parkinson’s disease, postural instability, rehabilitation

## Abstract

**Background:**

Postural instability and impaired balance are disabling, levodopa-resistant features of Parkinson’s disease (PD) and leading contributors to falls. Many systematic reviews and meta-analyses (SR/MAs) report that exercise improves balance, but they differ in modality, comparator, instrument and rigor and have not been synthesized and graded as a whole. We conducted an umbrella review of meta-analytic evidence on exercise and balance in PD.

**Methods:**

Following PRISMA 2020 and umbrella-review methodology, PubMed and Web of Science were searched from inception to 8 June 2026. Pairwise SR/MAs of randomized controlled trials (RCTs) reporting balance performance (Berg Balance Scale [BBS], Mini-BESTest), mobility (Timed Up-and-Go [TUG]) or falls in PD were eligible; network meta-analyses were supporting evidence only. We extracted the pooled random-effects estimates as reported by each review (no re-pooling of individual-trial data), harmonized their direction, and interpreted standardized effects as small/moderate/large. Methodological quality, primary-study overlap and certainty were appraised with AMSTAR-2, the corrected covered area (CCA) and GRADE.

**Results:**

From 161 unique records, 20 pairwise meta-analyses were included, spanning aerobic, resistance, aquatic, dance, VR/exergaming, traditional Chinese exercise, and home-based and group formats. Exercise improved balance in most modalities, though not universally (resistance training and one mixed-exercise review were non-significant). Because estimates came from separate meta-analyses, they are not directly comparable; standardized balance-performance effects ranged from small (g = 0.33, 95% CI 0.11–0.55) to large (aerobic, BBS g = 0.99, 0.76–1.23). Evidence on falls was inconsistent (proportion of fallers unchanged; fall rate reduced). Between-review overlap was very high at the BBS node (CCA = 16.8%). AMSTAR-2 confidence was moderate in 9, low in 10 and critically low in 1; GRADE certainty was moderate-to-very-low.

**Conclusion:**

Exercise is associated with small-to-large improvements in clinical balance in PD, but evidence on falls is inconsistent, and the underlying meta-analyses are mostly of low-to-moderate quality, highly overlapping, and of moderate-to-very-low certainty. Aerobic, mind–body and game-based modalities show relatively consistent signals, but this umbrella review cannot establish superiority of any modality. Larger, blinded, registered RCTs with standardized balance and prospectively ascertained fall outcomes are needed.

## Background

Parkinson’s disease (PD) is the fastest-growing neurological disorder worldwide, affecting more than six million people, with prevalence expected to more than double by 2040 owing to population aging (GBD 2016 Parkinson’s Disease Collaborators, 2016). It is characterized neuropathologically by progressive degeneration of dopaminergic neurons in the substantia nigra pars compacta and the accumulation of α-synuclein-containing Lewy bodies, producing the cardinal motor features of bradykinesia, rigidity, rest tremor and, in later stages, postural instability (Bloem et al., 2021). Among these, postural instability and balance impairment are particularly disabling because they respond poorly to dopaminergic medication and to deep-brain stimulation, and they are among the strongest determinants of falls, fall-related injury, fear of falling, reduced mobility and loss of independence (Kim et al., 2013). Up to two-thirds of people with PD fall each year, and balance dysfunction is a principal driver of institutionalization and diminished quality of life (Allen et al., 2013). It is important to note at the outset that balance performance and falls are related but distinct: balance tests quantify postural or functional performance, whereas a fall is a clinical event that depends additionally on cognition, freezing of gait, orthostatic hypotension, medication, environment and activity exposure (Kim et al., 2013; Allen et al., 2013).

Because the postural-control deficits of PD arise from a combination of impaired proprioceptive integration, deficient anticipatory postural adjustments, axial rigidity and slowed reactive responses, they are amenable to activity-dependent and task-specific rehabilitation (Mak et al., 2017). Exercise has therefore become a cornerstone of non-pharmacological management. Several mechanisms have been proposed—experience-dependent neuroplasticity, upregulation of brain-derived neurotrophic factor, enhanced dopaminergic and corticostriatal signaling, and improved cerebellar and sensorimotor integration, alongside peripheral adaptations in muscle strength, flexibility and cardiorespiratory fitness (Petzinger et al., 2013; Schenkman et al., 2018)—but these mechanisms are hypotheses derived from separate experimental work and were not evaluated by the clinical reviews synthesized here. They nonetheless motivate a wide range of modalities, from aerobic and resistance training to aquatic therapy, dance, virtual reality (VR)/exergaming, and mind–body practices such as Tai Chi, Qigong and Baduanjin.

Reflecting this breadth, the past 15 years have produced a large and rapidly expanding number of SR/MAs examining the effect of exercise on balance in PD. Individually, these reviews differ in the modalities compared, the comparator used (no intervention, usual care or active control), the balance instrument analyzed (Berg Balance Scale [BBS], Mini-Balance Evaluation Systems Test [Mini-BESTest], Functional Reach or composite balance-related-activity scales; the Timed Up-and-Go [TUG] as a mobility index), the effect metric reported, and their methodological rigor. The resulting picture is fragmented and at times contradictory, and the aggregate strength, credibility and quality of the evidence have not been formally synthesized.

An umbrella review—a systematic synthesis of existing SR/MAs—is the appropriate design to address this gap (Aromataris et al., 2015; Fusar-Poli and Radua, 2018). By collating, re-appraising and grading the meta-analytic evidence, an umbrella review can map which modalities have been studied, quantify between-review overlap and heterogeneity, and expose where the evidence is robust versus fragile; it cannot, however, establish the comparative superiority of one modality over another, because that requires direct (head-to-head) evidence. A recent umbrella review in this journal evaluated VR training across motor, activity-of-daily-living and quality-of-life outcomes in PD (Yu et al., 2023), and another summarized “unconventional” exercises for PD motor function (Zhang et al., 2025), but none has focused specifically on balance—the outcome most tightly linked to falls and disability—across the full spectrum of exercise modalities. The aim of the present umbrella review was therefore to systematically identify, re-appraise and grade SR/MAs of RCTs evaluating the effect of exercise on balance in people with PD, and to describe the magnitude, consistency and certainty of the evidence for each modality.

## Methods

This umbrella review was designed and reported in accordance with the PRISMA 2020 statement (Page et al., 2021) and with established guidance for the conduct and reporting of umbrella reviews (Aromataris et al., 2015; Fusar-Poli and Radua, 2018; Pollock et al., 2024). The completed PRISMA 2020 checklist is provided as Supplementary file 1.

### Eligibility criteria and outcome definitions

Eligibility followed the PICOS framework (Table 1). We included pairwise SR/MAs of RCTs that (i) enrolled adults with idiopathic PD; (ii) evaluated a structured exercise intervention of any modality; (iii) used a non-exercise comparator (no intervention, usual care, sham/placebo) or, where explicitly stated, an active exercise comparator; (iv) reported at least one quantitative balance-related outcome with a pooled estimate; and (v) pooled at least two RCTs. We prespecified three outcome domains and analyzed and interpreted them separately: (a) balance-performance measures (BBS, Mini-BESTest, Functional Reach, composite balance-related-activity scales); (b) mobility, indexed by the TUG, which reflects dynamic balance together with gait initiation, turning and lower-limb strength and is therefore not a pure balance test; and (c) falls, the principal clinical consequence of balance impairment, retained as a secondary, clinically-anchored outcome. Interventions were classified by exercise type; “home-based” and “group” denote the mode of delivery rather than a distinct exercise modality and were treated accordingly. We excluded narrative reviews, primary RCTs, protocols and reviews of non-idiopathic parkinsonism. Because their indirect estimates are not comparable with pairwise random-effects estimates, network meta-analyses (NMAs) were not part of the primary synthesis but were retained as supporting evidence (Supplementary file 4).

**Table 1:** Eligibility criteria (PICOS).

Element	Inclusion	Exclusion
Population	Adults with idiopathic Parkinson’s disease (any Hoehn & Yahr stage)	Atypical/secondary parkinsonism; mixed neurological samples not reported separately
Intervention	Structured exercise of any modality (aerobic, resistance, aquatic, dance, VR/exergaming, Tai Chi/Qigong/Baduanjin); home-based and group formats treated as delivery modes	Pharmacological, surgical or passive interventions without an exercise component
Comparator	No intervention, usual care, sham/placebo (primary); active exercise where explicitly stated	—
Outcome	Balance-performance (BBS, Mini-BESTest, Functional Reach, balance-related-activity scales), mobility (TUG), or falls, with a pooled estimate	Reviews without a quantitative balance-related estimate
Study design	Systematic review with pairwise meta-analysis of RCTs (≥ 2 RCTs pooled)	Narrative reviews; primary studies; protocols; network meta-analyses (retained only as supporting evidence)

BBS Berg Balance Scale, TUG Timed Up-and-Go, VR virtual reality, RCT randomized controlled trial.

### Information sources and search strategy

PubMed and Web of Science were searched from inception to 8 June 2026. PubMed was searched with (exercise [Title/Abstract]) AND (Parkinson’s [Title/Abstract]); Web of Science was searched with Parkinson’s (Title) AND exercise (Title) AND meta (Title). The search was designed by the review team and executed independently by two reviewers (YYS, SYX), who cross-checked the retrieved records; a professional information specialist/librarian was not consulted. The strategy was intentionally high-precision rather than high-sensitivity: PubMed provides broad biomedical indexing and Web of Science broad citation coverage, and to mitigate the risk of missing eligible reviews we hand-searched the reference lists of all included reviews and of two prior PD umbrella reviews (Yu et al., 2023; Zhang et al., 2025). We acknowledge that this two-database strategy, the restriction of the Web of Science query to titles containing “meta,” and the absence of controlled-vocabulary synonyms for physical activity, rehabilitation, balance and systematic review are likely to have reduced recall and may have missed reviews indexed only in Embase, Scopus, the Cochrane Library, PEDro or SPORTDiscus; this is discussed as a limitation. The full, line-by-line strategies and dates are provided in Supplementary file 2. Records were imported into Zotero for de-duplication. No language restriction was applied at screening.

### Study selection

After de-duplication and removal of retracted articles and duplicate publications, two reviewers independently screened titles/abstracts and then full texts against the eligibility criteria; disagreements were resolved by a third reviewer. For the four reports that could not be retrieved, we searched institutional and open-access repositories and attempted to obtain them through interlibrary services; they remained unavailable and were excluded, and we judged their omission unlikely to alter the synthesis given the breadth of the retained evidence. When two or more eligible reviews addressed the same modality–outcome pair, a pre-specified rule retained the single most informative review, based on (in order) the largest number of contributing RCTs, the most recent search date and the highest AMSTAR-2 rating; superseded reviews were recorded as overlap-excluded and used only to corroborate findings (Fusar-Poli and Radua, 2018).

### Data extraction, harmonization and synthesis

Two reviewers independently extracted, into a piloted form, the first author, year, search end-date, PD population and stage, exercise modality and dose, comparator, number of contributing RCTs, participants, outcome and instrument, the pooled effect estimate with 95% CI, heterogeneity (I^2^), any reported prediction interval, the analysis model and the risk-of-bias tool used by the primary review; discrepancies were resolved by consensus. Importantly, this umbrella review did not re-pool individual-trial data or recompute effect sizes *de novo*. For every association we used the pooled random-effects estimate exactly as reported by the primary review. To aid readability we harmonized the direction of effects (so that a positive value denotes better balance and a negative TUG value denotes faster, i.e., better, performance) and, where a review reported a standardized mean difference (Hedges’ g/SMD), we tabulated it as such; where a review reported an instrument-anchored mean difference (MD; e.g., BBS points), we retained the MD without converting it. We did not transform MDs into SMDs or vice versa. Standardized effects were interpreted descriptively as small (≈ 0.2), moderate (≈ 0.5) or large (≥ 0.8) (DerSimonian and Laird, 1986); because these thresholds and the minimal clinically important difference (MCID) are instrument-specific, MCID benchmarks (≈ 5 BBS points; ≈ 3.5 s for TUG) (Steffen and Seney, 2008) were applied only to instrument-anchored MDs and never to standardized effects. The between-study variance estimator (predominantly DerSimonian–Laird) and any prediction interval or Hartung–Knapp–Sidik–Jonkman (HKSJ) adjustment were those used by the primary reviews; we report them where available but did not apply them ourselves (Riley et al., 2011; IntHout et al., 2014). Statistical heterogeneity, where reported, is presented as I^2^ and interpreted as low (< 25%), moderate (25–75%) or high (> 75%) (Higgins et al., 2003).

### Reporting-bias considerations

Because we did not have access to harmonized study-level data across reviews, we did not perform new tests of small-study effects or an excess-significance test (Ioannidis and Trikalinos, 2007). Instead, we report small-study-effect and publication-bias assessments [e.g., funnel plots, Egger’s test (Egger et al., 1997)] as conducted and reported by the primary reviews, and we interpret them cautiously: formal testing was available for only a minority of associations, and the small number of trials per review limits statistical power, so we draw no firm conclusion about the presence or absence of publication bias.

### Methodological quality (AMSTAR-2)

The methodological quality of each included review was independently rated by two reviewers using AMSTAR-2, which comprises 16 items, seven of which are critical (items 2, 4, 7, 9, 11, 13, 15) (Shea et al., 2017). Overall confidence was classified as high, moderate, low or critically low according to the number and type of weaknesses. Disagreements were resolved by consensus. The full item-level appraisal is provided in Supplementary file 5.

### Overlap of primary studies (corrected covered area)

Because included reviews frequently share primary RCTs, overlap was quantified with the corrected covered area (CCA = (N − r) / (r × c − r), where N is the number of primary-study occurrences counting repeats, r the number of distinct primary studies and c the number of reviews contributing to a node), interpreted as slight (0–5%), moderate (6–10%), high (11–15%) or very high (> 15%) (Pieper et al., 2014). CCA was computed per outcome node, and both the highest and lowest node values are reported. The overlap matrix is provided in Supplementary file 6.

### Certainty of evidence (GRADE)

For each association, two reviewers independently rated certainty using GRADE (Guyatt et al., 2008). Because GRADE was developed to rate a body of evidence rather than a meta-analysis as a unit, we applied it at the level of each modality–outcome association and treated each included review as the best-available body of RCT evidence for that association, judging the five domains as follows: risk of bias, from the risk-of-bias assessment reported by the primary review; inconsistency, from I^2^ and the width/overlap of CIs; indirectness, from the fit of the population, intervention and outcome to our question (TUG, being a mobility index, was flagged as indirect for balance); imprecision, from CI width and total sample size; and publication bias, from what the primary review reported. Crucially, because of the very high primary-study overlap, we did not treat agreement between reviews that shared the same trials as independent replication and therefore did not upgrade certainty on the basis of consistency across overlapping reviews. Disagreements were resolved by consensus; per-association judgments are reported in Supplementary file 3.

## Results

### Study identification and selection

The search retrieved 221 records (PubMed *n* = 121; Web of Science *n* = 100). After removing 53 duplicates, 3 retracted articles and 4 duplicate publications, 161 unique records were screened. Title/abstract screening excluded 96 records; 65 reports were sought, of which 4 could not be retrieved, leaving 61 assessed in full. Of these, 41 were excluded (18 not a meta-analysis; 9 with no quantitative balance-related outcome; 7 network meta-analyses retained only as supporting evidence; 7 superseded by a more comprehensive overlapping review). Twenty pairwise meta-analyses met all criteria and were included (Figure 1); the seven supporting NMAs (Qian et al., 2023; Wang et al., 2023; Lorenzo-García et al., 2024; Gao et al., 2024; Fan et al., 2025; Mao et al., 2025; Tong et al., 2025) are listed in Supplementary file 4.

**Figure 1:**
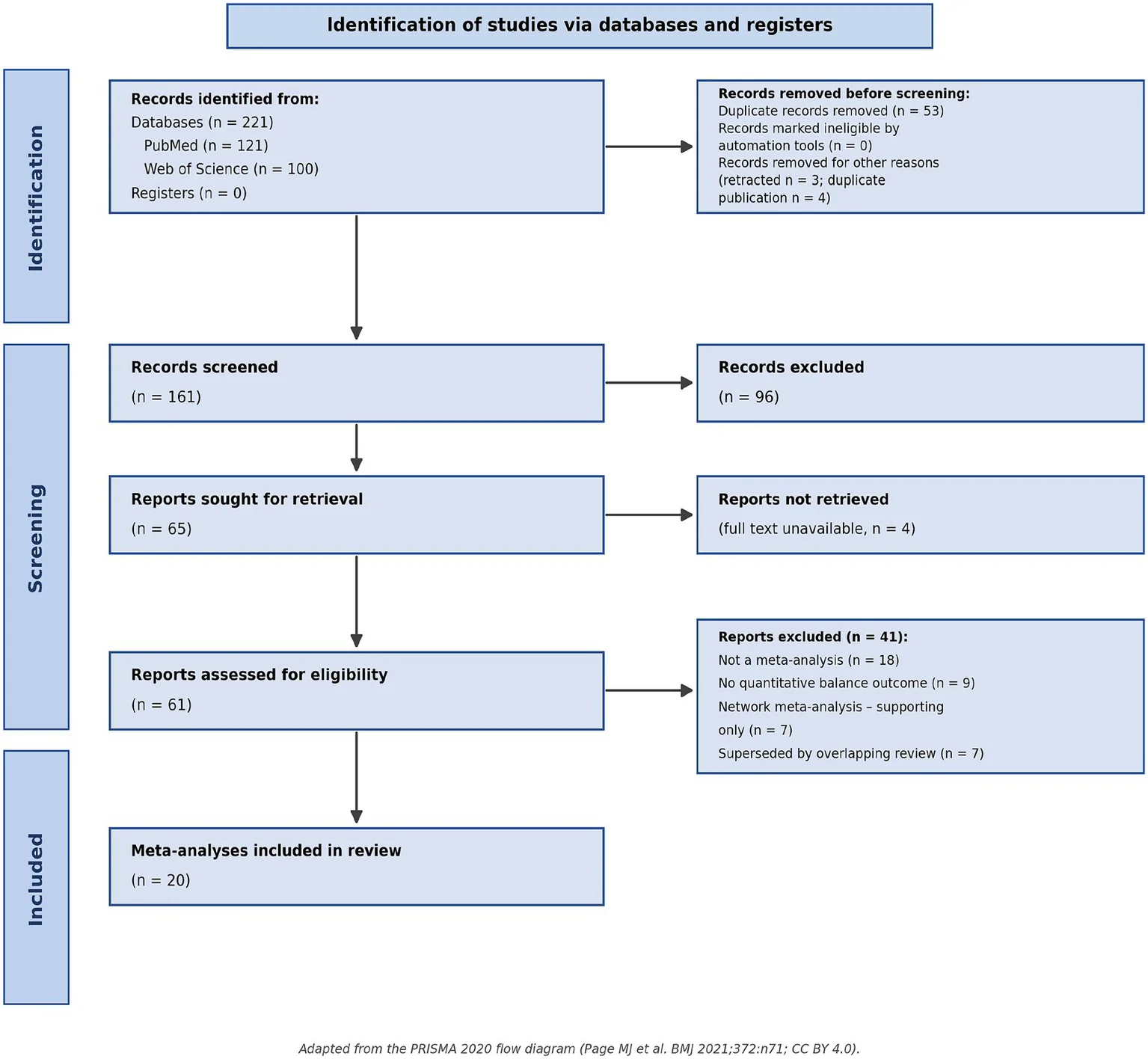
PRISMA 2020 flow diagram (databases and registers). Template: Page et al. (2021) (CC BY 4.0).

### Characteristics of included reviews

The 20 included meta-analyses were published between 2011 and 2026. Individually they pooled between 5 and 62 RCTs (median ≈ 15) and, where reported, between 380 and 3,274 participants; reported intervention durations were typically 4–12 weeks. They spanned the full range of exercise types: general/mixed exercise (Allen et al., 2011; Shen et al., 2016; Li et al., 2025; Park et al., 2025), aerobic exercise (Zhen et al., 2022), aerobic plus resistance training (Song et al., 2025), resistance training (Gollan et al., 2022), traditional Chinese exercise (Tai Chi, Qigong, Baduanjin) (Wu et al., 2022; Lou et al., 2025; Du et al., 2025; Yuan and Wang, 2025; Lai et al., 2022), dance (Sharp and Hewitt, 2014; Dos Santos Delabary et al., 2018), aquatic/water-based exercise (Gomes Neto et al., 2020; Braz de Oliveira et al., 2024), and VR/exergaming (De Natale et al., 2025; Hernandez-Martinez et al., 2025); two further reviews addressed the mode of delivery—group (States et al., 2025) and home-based (Flynn et al., 2019) exercise—rather than a distinct modality. The most frequently analyzed balance-performance instrument was the BBS, followed by the Mini-BESTest; the TUG was the commonest mobility index. Characteristics and pooled estimates are summarized in Table 2, with per-association data (including the seven supporting NMAs) in Supplementary files 3, 4.

**Table 2:** Characteristics and pooled balance-related effects of the 20 included meta-analyses.

Review (year)	Modality (delivery)	k/N	Domain — instrument	Pooled effect (95% CI) as reported	I^2^	AMSTAR-2
Allen et al. (2011)	Mixed exercise & motor training	15 / —	Balance — activity (g)	0.33 (0.11, 0.55)	NR	Low
Allen et al. (2011)	Mixed exercise & motor training	2 / —	Falls — proportion of fallers (RR)	1.02 (0.66, 1.58)	NR	Low
Shen et al. (2016)	Mixed exercise	24/12*	Balance & gait (g)	0.30 ST; 0.42 LT (*p* < 0.05)	NR	Moderate
Shen et al. (2016)	Mixed exercise	4/5*	Falls — fall rate (RaR)	0.485 ST; 0.413 LT (P < 0.05)	NR	Moderate
Li et al. (2025)	Mixed exercise	62/3,274	Balance (SMD)	0.53 (0.33, 0.74)	NR	Moderate
Li et al. (2025)	Mixed exercise	62/3,274	Mobility — TUG (SMD)	−0.44 (−0.60, −0.28)	NR	Moderate
Park et al. (2025)	Exercise (older adults ≥60)	11/388	Balance (SMD)	−0.06 (*p* = 0.41, NS)	NR	Low
Zhen et al. (2022)	Aerobic exercise	20 / —	Balance — BBS (SMD)	0.99 (0.76, 1.23)	NR	Moderate
Zhen et al. (2022)	Aerobic exercise	20 / —	Mobility — TUG (SMD)	−0.41 (−0.61, −0.22)	NR	Moderate
Song et al. (2025)	Aerobic training	15/792	Balance — Mini-BESTest (SMD)	2.12 (−0.70, 4.94), NS	90%	Low
Song et al. (2025)	Resistance training	15/792	Mobility — TUG (SMD)	−0.03 (−1.60, 1.54), NS	63%	Low
Gollan et al. (2022)	Resistance training	18 / —	Balance & mobility	Non-significant	NR	Moderate
Wu et al. (2022)	Traditional Chinese exercise	15 / —	Balance — BBS (g)	0.83 (0.37, 1.29)	84%	Moderate
Wu et al. (2022)	Traditional Chinese exercise	15 / —	Mobility — TUG (g)	−0.80 (−1.13, −0.47)	81%	Moderate
Lou et al. (2025)	Tai Chi	16/963	Balance — function (MD)	2.06 (1.35, 2.78)	0%	Low
Du et al. (2025)	Tai Chi / Chinese health ex.	18/950	Balance — BBS (MD, points)	2.79 (2.44, 3.14)	NR	Low
Yuan and Wang (2025)	Traditional Chinese exercise	14/833	Balance — BBS (MD, points)	3.68 (2.09, 5.27)	NR	Low
Lai et al. (2022)	Baduanjin	10/804	Balance & gait	Significant (favors ex.)	mod	Moderate
Sharp and Hewitt (2014)	Dance	5 / —	Balance — BBS (SMD)	0.72 (0.31, 1.44)	NR	Critically low
Dos Santos Delabary et al. (2018)	Dance	NR	Mobility — TUG (MD)	Significant (favors dance)	NR	Low
Gomes Neto et al. (2020)	Water-based exercise	15/435	Balance (MD)	Significant (favors ex.)	NR	Low
Braz de Oliveira et al. (2024)	Aquatic exercise	12/380	Balance — postural (SMD)	0.47 (0.07, 0.87)†	NR	Low
De Natale et al. (2025)	Virtual reality	11/518	Balance — BBS (SMD)	Significant (favors VR)	NR	Moderate
Hernandez-Martinez et al. (2025)	Exergaming	14/406	Balance — BBS (ES)‡	0.90 (*p* < 0.01)	NR	Moderate
States et al. (2025)	Group exercise (delivery)	27/1,200	Balance — Mini-BEST / BBS (MD)	2.72 (1.88, 3.57); 4.31 (1.33, 7.29)	NR	Moderate
States et al. (2025)	Group exercise (delivery)	27/1,200	Mobility — TUG (MD)	−2.29 (−3.56, −1.02)	NR	Moderate
Flynn et al. (2019)	Home-based exercise (delivery)	NR	Balance — activities	Significant (favors ex.)	NR	Low

k contributing RCTs, N participants, NR not reported, NS non-significant, SMD standardized mean difference, MD mean difference, ES effect size, RR risk ratio, RaR rate ratio, ST short-term, LT long-term, BBS Berg Balance Scale, TUG Timed Up-and-Go. Estimates are reported exactly as in the primary review; where a review gave only the direction/significance of a pooled estimate (no extractable SMD/MD with CI), that is stated. *Shen et al. (2016) pooled 24/12 (balance) and 4/5 (falls) short−/long-term datasets. †Braz de Oliveira et al. (2024) CI derived from the reported SMD and *p*-value (full text unavailable). ‡Hernandez-Martinez et al. (2025) compared exergaming with active exercise; no CI was reported. Positive g/SMD/MD on balance scales = better balance; negative SMD/MD on TUG = better (faster) mobility. TUG is a mobility index, not a pure balance test.

### Effects of exercise on balance performance, by modality

The estimates below come from separate meta-analyses that differ in population, comparator, dose, disease stage and instrument; they therefore describe the evidence available for each modality and must not be read as head-to-head comparisons or as demonstrating that one modality is superior to another. With that caveat, exercise improved balance performance in most—but not all—modalities. Among the broad mixed-exercise syntheses, effects were small to moderate: Allen et al. (2011) reported a Hedges’ g of 0.33 (95% CI 0.11–0.55) across 15 RCTs, Shen et al. (2016) reported g = 0.30 (short-term) and 0.42 (long-term), and the largest synthesis, Li et al. (2025), reported g = 0.53 (0.33–0.74) across 62 RCTs. In contrast, Park et al. (2025) found no significant balance effect in older adults (SMD = −0.06, *p* = 0.41), despite a large gain in gait speed.

Where a standardized balance-performance estimate with a CI was available (Figure 2), the point estimate was numerically largest for aerobic exercise (Zhen et al., 2022, BBS SMD 0.99, 0.76–1.23) and traditional Chinese exercise (Wu et al., 2022, BBS g 0.83, 0.37–1.29), followed by dance (Sharp and Hewitt, 2014, 0.72, 0.31–1.44), mixed exercise (Li et al., 2025, 0.53), aquatic exercise (Braz de Oliveira et al., 2024, 0.47, 0.07–0.87) and Allen’s mixed-exercise estimate (0.33). Again, these magnitudes cannot be ranked as evidence of comparative efficacy. Exergaming produced a large BBS effect relative to conventional exercise (Hernandez-Martinez et al., 2025, ES = 0.90; no CI reported), and VR improved the BBS versus conventional therapy (De Natale et al., 2025).

**Figure 2:**
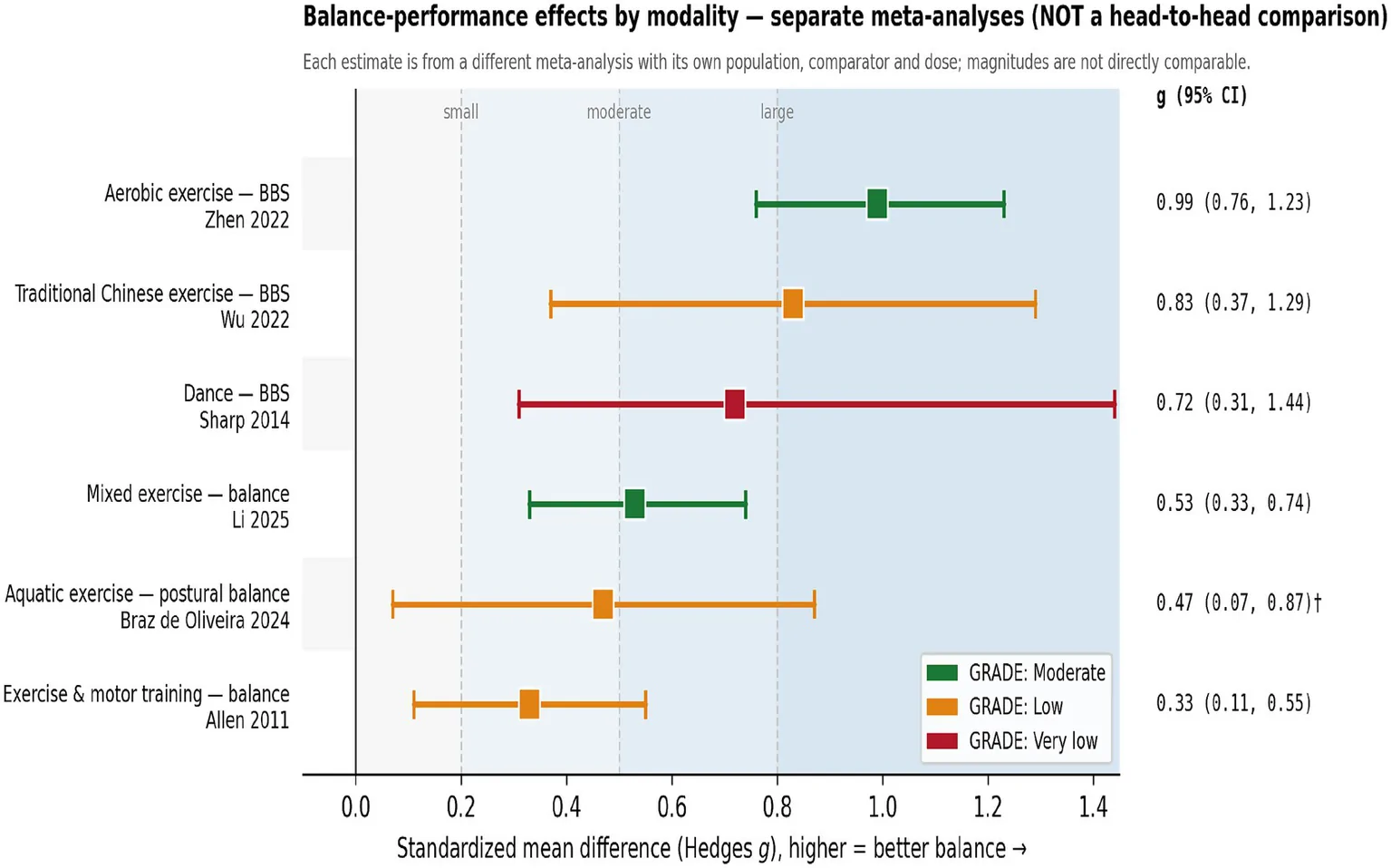
Balance-performance effects (Hedges’ g/SMD) by modality, each from a separate meta-analysis. This is NOT a head-to-head comparison: every estimate has its own population, comparator and dose, and magnitudes are not directly comparable. Only reviews reporting a standardized effect with a 95% CI are shown (instrument-anchored MD reviews and estimates without a CI are in Table 2). Squares = point estimates; whiskers = 95% CIs; color = GRADE certainty; a higher value = better balance. †Braz de Oliveira et al. (2024) CI derived from the reported SMD and *p*-value.

Instrument-anchored mind–body reviews were consistent on the BBS in points: Yuan and Wang (2025) MD = 3.68 (2.09–5.27), Du et al. (2025) MD = 2.79 (2.44–3.14) and Lou et al. (2025) balance MD = 2.06 (1.35–2.78, I^2^ = 0%). Interpreted against the ≈ 5-point BBS MCID (a comparison valid only for these point-based MDs), these gains approach but do not all reach the clinically important threshold. Water-based exercise improved balance in Gomes Neto et al. (2020) (mean difference favoring exercise) and by an SMD of 0.47 in Braz de Oliveira et al. (2024). For the delivery-defined reviews, group exercise (States et al., 2025) improved the Mini-BESTest (MD 2.72, 1.88–3.57) and BBS (MD 4.31, 1.33–7.29) but only 3 of 7 balance measures reached significance, and home-based exercise improved balance-related activities (Flynn et al., 2019); a supporting network meta-analysis of home-based exercise (Gao et al., 2024) estimated a small balance effect (SMD 0.18, 0.04–0.33).

Not all modalities showed benefit. Isolated resistance training was non-significant for balance and mobility (Gollan et al., 2022; Song et al., 2025, TUG SMD = −0.03, −1.60 to 1.54), the aerobic Mini-BESTest estimate in Song et al. (2025) was large but very imprecise and highly heterogeneous (SMD 2.12, −0.70 to 4.94; I^2^ = 90%), crossing the null, and boxing produced no significant balance benefit (non-significant Hedges’ g) in a separate synthesis. Thus the magnitude and precision of balance effects differed markedly across the evidence base, and several estimates were non-significant or imprecise.

### Effects on mobility (TUG) and falls

Mobility, indexed by the TUG, improved with mixed exercise (Li et al., 2025, SMD − 0.44, −0.60 to −0.28), aerobic exercise (Zhen et al., 2022, −0.41, −0.61 to −0.22), traditional Chinese exercise (Wu et al., 2022, −0.80, −1.13 to −0.47) and group exercise (States et al., 2025, MD − 2.29 s, −3.56 to −1.02), but not with isolated resistance training (Song et al., 2025). Because the TUG incorporates gait, turning and lower-limb strength, these gains should be read as improvements in functional mobility that only partly reflect balance.

Evidence on falls was inconsistent and, importantly, depended on how falls were measured. Allen et al. (2011) found no effect on the proportion of fallers (i.e., the risk of falling at least once; risk ratio 1.02, 95% CI 0.66–1.58, 2 trials), whereas Shen et al. (2016) found a reduced fall rate (i.e., the number of falls per person-time; rate ratio 0.485 short-term and 0.413 long-term, both *p* < 0.05). These are distinct quantities—a binary risk versus an event rate—estimated with different statistical models, over different follow-up durations and with different fall-ascertainment methods (prospective diaries versus retrospective recall), which can readily produce apparently contradictory conclusions. Reviews of mind–body and group exercise did not report consistent effects on the Falls Efficacy Scale (fear of falling). Overall, exercise improved balance-test scores more consistently than it reduced falls, a dissociation examined in the Discussion.

### Heterogeneity and prediction intervals

Heterogeneity, where reported, was variable. It was negligible for some instrument-anchored mind–body estimates (Lou et al., 2025, I^2^ = 0%) but high for several standardized estimates (Wu et al., 2022, BBS I^2^ = 84% and TUG I^2^ = 81%; Song et al., 2025, Mini-BESTest I^2^ = 90%; Song et al., 2025, resistance-TUG I^2^ = 63%). Many reviews did not report I^2^ for every outcome, and few reported prediction intervals; consequently a complete, uniform heterogeneity table could not be constructed from the primary reports, which is itself a limitation of the underlying evidence. Where prediction intervals were reported by the primary reviews they were substantially wider than the corresponding CIs and, for several modalities, included the null, indicating that a future trial could plausibly show no benefit. Potential sources of the observed heterogeneity that are identifiable from the included reviews include differences in Hoehn & Yahr stage and disease duration, medication (on/off) state, intervention duration, frequency and intensity, degree of supervision and delivery setting, the comparator used (no intervention versus active control), baseline balance impairment, and—most pervasively—the balance instrument analyzed. Because few reviews reported subgroup analyses across these factors in a comparable way, we describe them qualitatively rather than formally partitioning heterogeneity.

### Methodological quality (AMSTAR-2)

Overall confidence was moderate in 9 reviews, low in 10 and critically low in 1 (Sharp and Hewitt, 2014); no review reached high confidence. The most methodologically robust review was States et al. (2025), which had a prospectively registered protocol and a revised Cochrane risk-of-bias (RoB 2) assessment but, like most reviews, did not provide a justified list of excluded studies (critical item 7) and so was rated moderate rather than high. The commonest critical weaknesses across the set were the absence of a pre-registered protocol (item 2), no justified list of excluded studies (item 7) and no formal assessment of the impact of risk of bias on the pooled estimate (item 13); the near-universal failure to report funding sources of the primary studies (item 10, non-critical) was also notable. Item-level ratings are in Supplementary file 5 and summarized in Table 3.

**Table 3:** AMSTAR-2 overall confidence.

Overall confidence	*n* reviews	Reviews
High	0	—
Moderate	9	Shen et al. (2016); Li et al. (2025); Zhen et al. (2022); Gollan et al. (2022); Wu et al. (2022); Lai et al. (2022); De Natale et al. (2025); Hernandez-Martinez et al. (2025); States et al. (2025)
Low	10	Allen et al. (2011); Park et al. (2025); Song et al. (2025); Lou et al. (2025); Du et al. (2025); Yuan and Wang (2025); Dos Santos Delabary et al. (2018); Gomes Neto et al. (2020); Braz de Oliveira et al. (2024); Flynn et al. (2019)
Critically low	1	Sharp and Hewitt (2014)

Total = 20. Critical domains = AMSTAR-2 items 2, 4, 7, 9, 11, 13, 15. Full item-level appraisal in Supplementary file 5.

### Overlap of primary studies

Overlap varied by outcome node. It was very high at the most populated node (BBS-based balance): CCA = 16.8% (*N* = 170 study occurrences across c = 8 reviews; r ≈ 78 distinct RCTs). At sparser nodes with fewer contributing reviews (e.g., Mini-BESTest and falls), fewer trials were shared and CCA was correspondingly lower, in the slight-to-moderate range (approximately 4–7%); thus CCA across nodes ranged from about 4 to 16.8%. Even at the sparser nodes, however, the same landmark PD exercise trials recur, so the reviews are not statistically independent and their apparent agreement is partly an artifact of repeatedly re-pooling a shared core of trials—a point we weight heavily when interpreting cross-modality consistency. The full overlap matrix is provided in Supplementary file 6.

### Certainty of evidence (GRADE)

GRADE certainty ranged from moderate to very low; no association reached high certainty. The associations rated moderate—the most credible—were the aerobic-exercise BBS benefit (Zhen et al., 2022), the mixed-exercise balance benefit (Li et al., 2025) and the group-exercise balance benefit (States et al., 2025), each resting on a comparatively large evidence base, a methodologically stronger review and a CI that excluded trivial effects. Most modality-specific associations were low or very low, downgraded principally for risk of bias in the contributing RCTs (frequent lack of blinding and allocation concealment), inconsistency (high I^2^ and, where reported, prediction intervals crossing the null) and imprecision (small samples, wide CIs). As pre-specified, agreement among reviews sharing the same trials was not treated as independent replication and did not raise certainty. Per-association judgments are in Supplementary file 3.

## Discussion

This umbrella review synthesized 20 meta-analyses of exercise and balance in PD. Across a heterogeneous evidence base, exercise was associated with improvements in clinical balance performance in most modalities, with standardized effects that were, individually, small to large; however, several estimates were non-significant or imprecise, evidence on falls was inconsistent, and the underlying reviews were mostly of low-to-moderate methodological quality, highly overlapping and of moderate-to-very-low certainty. These features temper what can be concluded, and in particular they preclude ranking modalities against one another.

A central interpretive point is that this review aggregates indirect evidence. Each pooled estimate arises from a different set of trials with its own population, comparator, dose and outcome instrument, so larger point estimates for aerobic, mind–body or game-based exercise cannot be read as demonstrating superiority over resistance or other training; establishing comparative efficacy requires head-to-head RCTs or, with caveats, network meta-analysis (summarized separately in Supplementary file 4). We therefore describe these modalities as showing relatively consistent and, in places, larger signals, rather than as “best” or “first-choice” interventions. The observation that task-oriented, dynamic and multisensory programs tend to show clearer balance signals than isolated strengthening is a hypothesis generated by—not proven by—this synthesis.

Mechanistically, exercise has been proposed to improve balance in PD through experience-dependent neuroplasticity, corticostriatal and cerebellar adaptation, and the differential transfer of task-specific practice to postural control (Petzinger et al., 2013; Schenkman et al., 2018). These mechanisms are biologically plausible and may explain the pattern of findings, but they were not measured by any included review, which assessed clinical outcomes only; they should therefore be regarded as context from the wider literature rather than as conclusions of this umbrella review. From a measurement-science standpoint, the dissociation between improved balance-test scores and inconsistent fall outcomes is informative: balance scales such as the BBS show ceiling effects in ambulant people with PD and may miss the reactive and anticipatory deficits most relevant to falling (Kim et al., 2013; Mak et al., 2017), and falls are multifactorial events. This argues for greater use of instrumented and perturbation-based balance assessment—wearable inertial sensors and posturography—which fall within the neuroengineering scope of this journal and can capture postural-control dimensions invisible to ordinal clinical scales.

Two methodological features deserve particular weight. First, the very high overlap at the BBS node (CCA 16.8%) means that the concordance among balance reviews is driven substantially by a shared core of frequently re-analyzed trials; apparent replication across modalities is therefore weaker than the number of reviews suggests. Second, most contributing RCTs were small and unblinded, heterogeneity was often high, and—where reported—prediction intervals frequently crossed the null; these features, not the umbrella method itself, are what constrain certainty. We did not re-pool trials or apply new small-study or HKSJ analyses, so our synthesis inherits both the strengths and the limitations of the primary reviews; we have been explicit about which estimates were reported as standardized effects and which as instrument-anchored mean differences, and we have benchmarked MCID only against the latter.

Clinically, the evidence supports offering regular exercise to improve balance in PD, individualized to preference, disease stage and access, and embedded within multifactorial fall-prevention rather than presented as a stand-alone fall-prevention measure. Given the indirect, mostly low-to-moderate-certainty evidence, no single modality can be recommended as superior; aerobic, mind–body and game-based programs are reasonable options with relatively consistent balance signals and good adherence and safety in home- and group-based delivery (States et al., 2025; Flynn et al., 2019), but the choice should remain pragmatic. Clinicians should not assume that better balance-test scores translate into fewer falls.

### Limitations

Limitations of this umbrella review (as distinct from limitations of the underlying trials) include: a deliberately high-precision search of only two databases (PubMed and Web of Science), without a librarian and without controlled-vocabulary synonyms, which is likely to have reduced recall despite reference-list hand-searching; reliance on pooled estimates as reported by the primary reviews, without re-pooling individual-trial data, so that some estimates could not be standardized and a derived CI was required for one aquatic review; the exclusion of network meta-analyses from the primary synthesis; and the retention of a few associations only as direction/significance because no extractable pooled estimate with a CI was reported. Limitations inherited from the primary evidence include the predominance of small, unblinded RCTs at moderate-to-high risk of bias; very high primary-study overlap, which undermines the independence of the reviews; incomplete reporting of I^2^ and prediction intervals; and marked heterogeneity of balance instruments and exercise doses, which complicates any pooling. Together these mean that the certainty of the evidence is constrained largely at source.

## Conclusion

Across a broad and current evidence base, exercise is associated with small-to-large improvements in clinical balance performance in people with PD, but these gains are not uniform across modalities, do not consistently translate into reduced falls, and rest on meta-analyses that are mostly of low-to-moderate methodological quality, highly overlapping and of moderate-to-very-low certainty. Aerobic, mind–body and game-based programs show relatively consistent balance signals, but because the evidence is indirect this umbrella review cannot establish that any modality is superior to another. Exercise should be offered to improve balance in PD as part of an individualized, multifactorial program, while stronger evidence—larger, blinded, pre-registered head-to-head RCTs using standardized and instrumented balance measures together with prospectively ascertained fall outcomes—is needed before firm, modality-specific recommendations can be made.

## Data Availability

The original contributions presented in the study are included in the article/Supplementary material, further inquiries can be directed to the corresponding author.

## Supplementary material

SUPPLEMENTARY FILE 1 Completed PRISMA 2020 checklist.

SUPPLEMENTARY FILE 2 Full database search strategies (PubMed; Web of Science) and dates.

SUPPLEMENTARY FILE 3 Characteristics and extracted effect estimates of the included reviews, with per-association GRADE judgments.

SUPPLEMENTARY FILE 4 Supporting network meta-analyses of exercise for balance/postural instability in PD.

SUPPLEMENTARY FILE 5 Item-level AMSTAR-2 appraisal of all included reviews.

SUPPLEMENTARY FILE 6 Corrected covered area (CCA) primary-study overlap matrix.

## Glossary

AMSTAR-2: A MeaSurement Tool to Assess systematic Reviews, version 2
BBS: Berg Balance Scale
CCA: corrected covered area
CI: confidence interval
ES: effect size
GRADE: Grading of Recommendations, Assessment, Development and Evaluations
HKSJ: Hartung–Knapp–Sidik–Jonkman
MCID: minimal clinically important difference
MD: mean difference
Mini-BESTest: Mini-Balance Evaluation Systems Test
NMA: network meta-analysis
NS: non-significant
PD: Parkinson’s disease
PI: prediction interval
RaR: rate ratio
RCT: randomized controlled trial
RR: risk ratio
SMD: standardized mean difference
SR/MA: systematic review and meta-analysis
TUG: Timed Up-and-Go
VR: virtual reality
