# Supplementary material for: Effects of exercise on balance in people with Parkinson’s disease: an umbrella review of systematic reviews and meta-analyses of randomized controlled trials
Source: Front Psychol. 2026 Oct 13;17:1939213. doi: 10.3389/fpsyg.2026.1939213 (PMC13710104; doi:10.3389/fpsyg.2026.1939213)
Supplement: Supplementary file 4 [file supplementary_file_2.docx]

# Supplementary file 2. Database search strategies and search methodology

*Umbrella review of exercise and balance in Parkinson’s disease. Searches were run from database inception to 8 June 2026, without language restriction at screening.*

## Search execution and team

The search was designed by the review team and executed independently by two reviewers (Youyou Song and Shiyan Xu)), who cross-checked all retrieved records. A professional information specialist/librarian was not consulted; this is acknowledged as a limitation. The strategy was intentionally high-precision rather than high-sensitivity. To mitigate the risk of missing eligible reviews, the reference lists of all included reviews and of two prior Parkinson’s disease umbrella reviews were hand-searched.

## PubMed (MEDLINE)

Search string: (exercise[Title/Abstract]) AND (Parkinson’s[Title/Abstract])

Date of search: 8 June 2026. Records retrieved: 121.

## Web of Science Core Collection

Search string: Parkinson’s (Title) AND exercise (Title) AND meta (Title)

Date of search: 8 June 2026. Records retrieved: 100.

Note on sensitivity: requiring “meta” in the Web of Science title, and the absence of controlled-vocabulary synonyms for Parkinson’s disease, exercise, physical activity, rehabilitation, balance/postural control and systematic review/meta-analysis, are likely to have reduced recall and may have excluded eligible reviews indexed only in Embase, Scopus, the Cochrane Library, PEDro or SPORTDiscus. Database expansion is recommended for any future update.

## De-duplication and screening (Zotero)

Combined records (n = 221) were imported into Zotero. Duplicates removed: 53. Retracted articles removed: 3. Duplicate publications removed: 4. Unique records screened: 161. Reports sought: 65; not retrieved: 4; assessed in full: 61.

## Eligibility outcome

Twenty pairwise meta-analyses reporting a quantitative balance-related outcome in PD met all PICOS criteria and were included; seven network meta-analyses were retained as supporting evidence (Supplementary file 4). Excluded at full text (n = 41): not a meta-analysis (18); no quantitative balance-related outcome (9); network meta-analysis, supporting only (7); superseded by an overlapping review (7).
